# 1-(4-Meth­oxy­phen­yl)ethane-1,2-diyl 1,1′-biphenyl-2,2′-dicarboxyl­ate

**DOI:** 10.1107/S160053681201848X

**Published:** 2012-05-05

**Authors:** Hoong-Kun Fun, Ching Kheng Quah, Dongdong Wu

**Affiliations:** a X-ray Crystallography Unit, School of Physics, Universiti Sains Malaysia, 11800 USM, Penang, Malaysia; b School of Chemistry and Chemical Engineering, Nanjing University, Nanjing 210093, People’s Republic of China

## Abstract

In the title mol­ecule, C_23_H_18_O_5_, the meth­oxy-substituted benzene ring makes dihedral angles of 65.12 (4) and 88.55 (4)° with the other two benzene rings. These two benzene rings form a dihedral angle of 45.70 (4)°. In the crystal, mol­ecules are linked into inversion dimers by pairs of weak C—H⋯O hydrogen bonds.

## Related literature

For the background to this study has been set out in the preceding paper, see: Fun *et al.* (2012 ▶). For the stability of the temperature controller used in the data collection, see: Cosier & Glazer (1986 ▶). For standard bond-length data, see: Allen *et al.* (1987 ▶). For the preparation, see: Wu *et al.* (2012 ▶).
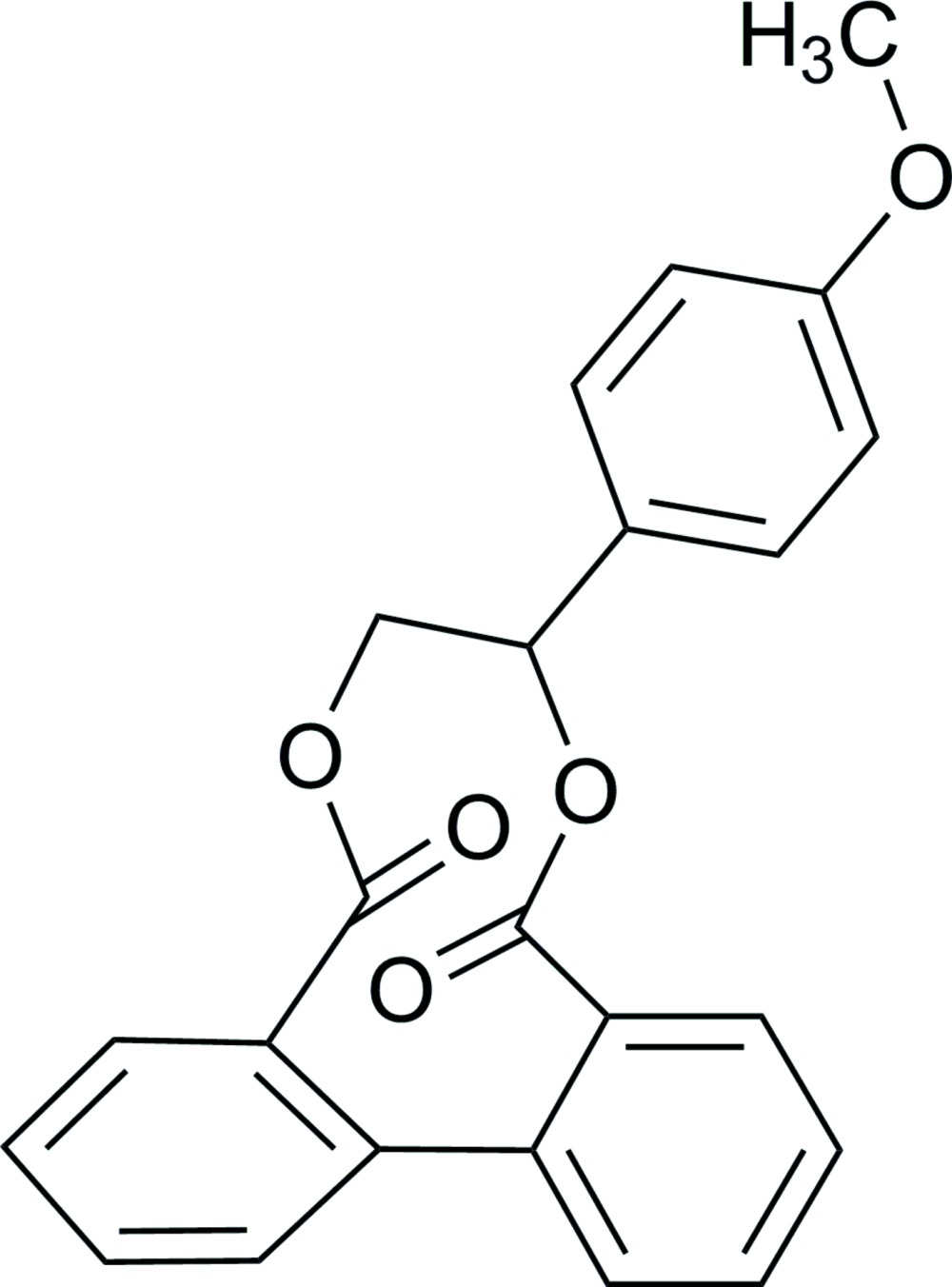


## Experimental

### 

#### Crystal data

C_23_H_18_O_5_
*M* *_r_* = 374.37
Monoclinic, 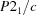

*a* = 14.0049 (5) Å
*b* = 10.8637 (4) Å
*c* = 12.7190 (5) Å
β = 110.150 (1)°
*V* = 1816.69 (12) Å^3^
*Z* = 4
Mo *K*α radiation
μ = 0.10 mm^−1^
*T* = 100 K
0.50 × 0.37 × 0.33 mm

#### Data collection

Bruker SMART APEXII DUO CCD area-detector diffractometer
Absorption correction: multi-scan (*SADABS*; Bruker, 2009 ▶) *T*_min_ = 0.954, *T*_max_ = 0.969
30595 measured reflections
8040 independent reflections
6872 reflections with *I* > 2σ(*I*)
*R*_int_ = 0.025

#### Refinement

*R*[*F* ^2^ > 2σ(*F* ^2^)] = 0.041
*wR*(*F* ^2^) = 0.122
*S* = 1.03
8040 reflections
254 parameters
H-atom parameters constrained
Δρ_max_ = 0.52 e Å^−3^
Δρ_min_ = −0.23 e Å^−3^

### 

Data collection: *APEX2* (Bruker, 2009 ▶); cell refinement: *SAINT* (Bruker, 2009 ▶); data reduction: *SAINT*; program(s) used to solve structure: *SHELXTL* (Sheldrick, 2008 ▶); program(s) used to refine structure: *SHELXTL*; molecular graphics: *SHELXTL*; software used to prepare material for publication: *SHELXTL* and *PLATON* (Spek, 2009 ▶).

## Supplementary Material

Crystal structure: contains datablock(s) global, I. DOI: 10.1107/S160053681201848X/is5123sup1.cif

Structure factors: contains datablock(s) I. DOI: 10.1107/S160053681201848X/is5123Isup2.hkl

Supplementary material file. DOI: 10.1107/S160053681201848X/is5123Isup3.cml

Additional supplementary materials:  crystallographic information; 3D view; checkCIF report

## Figures and Tables

**Table 1:** Hydrogen-bond geometry (Å, °)

*D*—H⋯*A*	*D*—H	H⋯*A*	*D*⋯*A*	*D*—H⋯*A*
C16—H16*A*⋯O3^i^	0.98	2.60	3.2676 (10)	126

Symmetry code: (i) 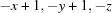.

**Fig. 1.**
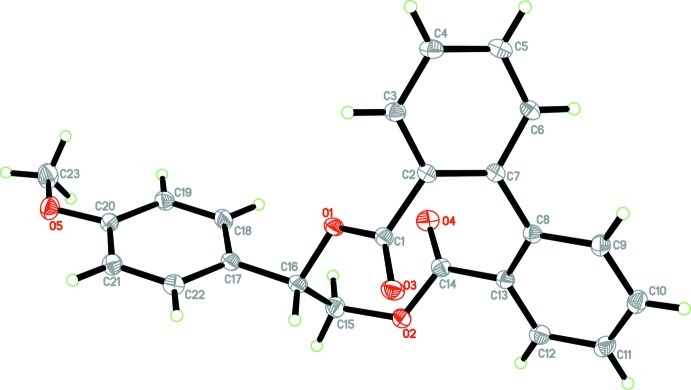
The molecular structure of the title compound, showing 50% probability displacement ellipsoids for non-H atoms.

**Fig. 2.**
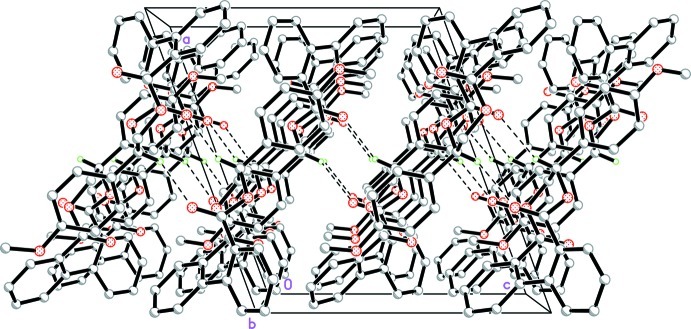
The crystal structure of the title compound, viewed along the b axis. H atoms not involved in hydrogen bonds (dashed lines) have been omitted for clarity.

## supplementary crystallographic information

### Comment

The title compound belongs to the biphenyl-containing bislactone family whose preparation could be achieved through a concise photochemical method (Wu *et al.*, 2012). In the title compound, Fig. 1, the methoxy attached phenyl ring (C17–C22) inclines at dihedral angles of 65.12 (4) and 88.55 (4)° with the two benzene rings (C2–C7 and C8–C13), respectively. The two benzene rings form a dihedral angle of 45.70 (4)°. Bond lengths (Allen *et al.*, 1987) and angles are within normal ranges and are comparable to a related structure (Fun *et al.*, 2012). In the crystal (Fig. 2), molecules are linked into inversion dimers by pairs of intermolecular C16—H16A···O3 hydrogen bonds (Table 1).

### Experimental

The title compound was the major diastereoisomer of the sequential photoreaction products of 9,10-phenanthrenedione with 1-methoxy-4-vinylbenzene. The compound was purified by flash column chromatography with ethyl acetate/petroleum ether (1:9) as eluents. X-ray quality crystals of the title compound were obtained from slow evaporation of an acetone and petroleum ether solution (1:10).

### Refinement

All H atoms were positioned geometrically and refined using a riding model with C—H = 0.93–0.98 Å and *U*_iso_(H) = 1.2 or 1.5*U*_eq_(C). A rotating-group model was applied for the methyl group.

### Crystal data

**Table:**

C_23_H_18_O_5_	*F*(000) = 784
*M**_r_* = 374.37	*D*_x_ = 1.369 Mg m^−^^3^
Monoclinic, *P*2_1_/*c*	Mo *K*α radiation, λ = 0.71073 Å
Hall symbol: -P 2ybc	Cell parameters from 9888 reflections
*a* = 14.0049 (5) Å	θ = 3.3–35.1°
*b* = 10.8637 (4) Å	µ = 0.10 mm^−^^1^
*c* = 12.7190 (5) Å	*T* = 100 K
β = 110.150 (1)°	Block, colourless
*V* = 1816.69 (12) Å^3^	0.50 × 0.37 × 0.33 mm
*Z* = 4	

### Data collection

**Table:**

Bruker SMART APEXII DUO CCD area-detector diffractometer	8040 independent reflections
Radiation source: fine-focus sealed tube	6872 reflections with *I* > 2σ(*I*)
Graphite monochromator	*R*_int_ = 0.025
φ and ω scans	θ_max_ = 35.1°, θ_min_ = 1.6°
Absorption correction: multi-scan (*SADABS*; Bruker, 2009)	*h* = −22→22
*T*_min_ = 0.954, *T*_max_ = 0.969	*k* = −17→16
30595 measured reflections	*l* = −20→20

### Refinement

**Table:**

Refinement on *F*^2^	Primary atom site location: structure-invariant direct methods
Least-squares matrix: full	Secondary atom site location: difference Fourier map
*R*[*F*^2^ > 2σ(*F*^2^)] = 0.041	Hydrogen site location: inferred from neighbouring sites
*wR*(*F*^2^) = 0.122	H-atom parameters constrained
*S* = 1.03	*w* = 1/[σ^2^(*F*_o_^2^) + (0.0706*P*)^2^ + 0.3453*P*] where *P* = (*F*_o_^2^ + 2*F*_c_^2^)/3
8040 reflections	(Δ/σ)_max_ = 0.001
254 parameters	Δρ_max_ = 0.52 e Å^−^^3^
0 restraints	Δρ_min_ = −0.23 e Å^−^^3^

### Special details

**Table:**

Experimental. The crystal was placed in the cold stream of an Oxford Cryosystems Cobra open-flow nitrogen cryostat (Cosier & Glazer, 1986) operating at 100.0 (1) K.
Geometry. All esds (except the esd in the dihedral angle between two l.s. planes) are estimated using the full covariance matrix. The cell esds are taken into account individually in the estimation of esds in distances, angles and torsion angles; correlations between esds in cell parameters are only used when they are defined by crystal symmetry. An approximate (isotropic) treatment of cell esds is used for estimating esds involving l.s. planes.
Refinement. Refinement of F^2^ against ALL reflections. The weighted R-factor wR and goodness of fit S are based on F^2^, conventional R-factors R are based on F, with F set to zero for negative F^2^. The threshold expression of F^2^ > 2sigma(F^2^) is used only for calculating R-factors(gt) etc. and is not relevant to the choice of reflections for refinement. R-factors based on F^2^ are statistically about twice as large as those based on F, and R- factors based on ALL data will be even larger.

### Fractional atomic coordinates and isotropic or equivalent isotropic displacement parameters (Å^2^)

**Table:**

	*x*	*y*	*z*	*U*_iso_*/*U*_eq_	
O1	0.38784 (4)	0.55150 (5)	0.14424 (4)	0.01591 (10)	
O2	0.35057 (4)	0.34260 (5)	0.01861 (5)	0.01878 (11)	
O3	0.35878 (4)	0.61128 (5)	−0.03585 (5)	0.01878 (11)	
O4	0.23791 (5)	0.35189 (5)	0.11150 (5)	0.02007 (11)	
O5	0.77642 (5)	0.51575 (6)	0.57884 (5)	0.02188 (12)	
C1	0.33248 (5)	0.60283 (6)	0.04471 (6)	0.01410 (11)	
C2	0.23425 (5)	0.64784 (6)	0.05242 (6)	0.01326 (11)	
C3	0.24048 (5)	0.74160 (6)	0.12951 (6)	0.01596 (12)	
H3A	0.3038	0.7721	0.1731	0.019*	
C4	0.15308 (6)	0.78980 (7)	0.14184 (7)	0.01838 (13)	
H4A	0.1576	0.8539	0.1918	0.022*	
C5	0.05903 (6)	0.74156 (7)	0.07911 (7)	0.01791 (13)	
H5A	0.0002	0.7724	0.0877	0.021*	
C6	0.05267 (5)	0.64697 (6)	0.00326 (6)	0.01502 (12)	
H6A	−0.0108	0.6148	−0.0377	0.018*	
C7	0.13932 (5)	0.59887 (6)	−0.01318 (6)	0.01314 (11)	
C8	0.12481 (5)	0.50195 (6)	−0.10053 (6)	0.01377 (11)	
C9	0.04937 (5)	0.52047 (7)	−0.20515 (6)	0.01666 (12)	
H9A	0.0091	0.5907	−0.2169	0.020*	
C10	0.03313 (6)	0.43626 (7)	−0.29216 (6)	0.01981 (13)	
H10A	−0.0186	0.4495	−0.3604	0.024*	
C11	0.09433 (6)	0.33242 (7)	−0.27690 (7)	0.02003 (13)	
H11A	0.0852	0.2775	−0.3356	0.024*	
C12	0.16915 (6)	0.31098 (7)	−0.17363 (7)	0.01812 (13)	
H12A	0.2103	0.2416	−0.1633	0.022*	
C13	0.18276 (5)	0.39331 (6)	−0.08526 (6)	0.01456 (11)	
C14	0.25758 (6)	0.36052 (6)	0.02653 (6)	0.01598 (12)	
C15	0.43566 (6)	0.34300 (7)	0.12253 (7)	0.02087 (14)	
H15A	0.4893	0.2904	0.1161	0.025*	
H15B	0.4146	0.3116	0.1825	0.025*	
C16	0.47489 (5)	0.47470 (7)	0.14896 (6)	0.01735 (12)	
H16A	0.5039	0.5029	0.0932	0.021*	
C17	0.55272 (5)	0.48569 (7)	0.26440 (6)	0.01667 (12)	
C18	0.53402 (6)	0.43948 (8)	0.35776 (7)	0.02047 (14)	
H18A	0.4718	0.4023	0.3484	0.025*	
C19	0.60657 (6)	0.44787 (8)	0.46475 (6)	0.01999 (13)	
H19A	0.5933	0.4160	0.5262	0.024*	
C20	0.69930 (5)	0.50455 (7)	0.47858 (6)	0.01664 (12)	
C21	0.71830 (6)	0.55306 (6)	0.38612 (6)	0.01783 (13)	
H21A	0.7798	0.5922	0.3957	0.021*	
C22	0.64573 (6)	0.54311 (6)	0.28016 (6)	0.01736 (12)	
H22A	0.6591	0.5750	0.2188	0.021*	
C23	0.76498 (7)	0.45148 (10)	0.67193 (7)	0.02796 (18)	
H23A	0.8262	0.4595	0.7357	0.042*	
H23B	0.7521	0.3660	0.6533	0.042*	
H23C	0.7089	0.4858	0.6892	0.042*	

### Atomic displacement parameters (Å^2^)

**Table:**

	*U*^11^	*U*^22^	*U*^33^	*U*^12^	*U*^13^	*U*^23^
O1	0.0142 (2)	0.0186 (2)	0.0154 (2)	0.00415 (17)	0.00568 (17)	0.00102 (17)
O2	0.0156 (2)	0.0204 (2)	0.0190 (2)	0.00381 (18)	0.00424 (18)	−0.00265 (18)
O3	0.0179 (2)	0.0224 (2)	0.0184 (2)	0.00192 (19)	0.00934 (19)	0.00145 (19)
O4	0.0244 (3)	0.0174 (2)	0.0200 (2)	0.00093 (19)	0.0096 (2)	0.00254 (18)
O5	0.0199 (3)	0.0270 (3)	0.0163 (2)	−0.0030 (2)	0.0032 (2)	−0.0001 (2)
C1	0.0129 (3)	0.0133 (2)	0.0162 (3)	0.0003 (2)	0.0052 (2)	−0.0004 (2)
C2	0.0133 (3)	0.0122 (2)	0.0153 (3)	0.00065 (19)	0.0062 (2)	0.00129 (19)
C3	0.0164 (3)	0.0141 (2)	0.0187 (3)	−0.0004 (2)	0.0078 (2)	−0.0016 (2)
C4	0.0202 (3)	0.0152 (3)	0.0227 (3)	0.0006 (2)	0.0112 (3)	−0.0023 (2)
C5	0.0175 (3)	0.0165 (3)	0.0229 (3)	0.0027 (2)	0.0110 (2)	0.0014 (2)
C6	0.0137 (3)	0.0149 (2)	0.0179 (3)	0.0014 (2)	0.0072 (2)	0.0027 (2)
C7	0.0135 (3)	0.0124 (2)	0.0144 (3)	0.00076 (19)	0.0058 (2)	0.00167 (19)
C8	0.0134 (3)	0.0141 (2)	0.0148 (3)	−0.00113 (19)	0.0061 (2)	0.0003 (2)
C9	0.0153 (3)	0.0190 (3)	0.0154 (3)	−0.0012 (2)	0.0049 (2)	0.0015 (2)
C10	0.0185 (3)	0.0242 (3)	0.0160 (3)	−0.0055 (2)	0.0050 (2)	−0.0011 (2)
C11	0.0206 (3)	0.0215 (3)	0.0189 (3)	−0.0072 (2)	0.0080 (3)	−0.0057 (2)
C12	0.0185 (3)	0.0157 (3)	0.0213 (3)	−0.0028 (2)	0.0084 (2)	−0.0038 (2)
C13	0.0143 (3)	0.0137 (2)	0.0163 (3)	−0.0012 (2)	0.0061 (2)	−0.0007 (2)
C14	0.0170 (3)	0.0120 (2)	0.0189 (3)	0.0012 (2)	0.0062 (2)	−0.0005 (2)
C15	0.0183 (3)	0.0196 (3)	0.0211 (3)	0.0057 (2)	0.0022 (3)	−0.0017 (2)
C16	0.0142 (3)	0.0214 (3)	0.0165 (3)	0.0049 (2)	0.0053 (2)	−0.0004 (2)
C17	0.0143 (3)	0.0199 (3)	0.0160 (3)	0.0029 (2)	0.0054 (2)	0.0003 (2)
C18	0.0143 (3)	0.0304 (4)	0.0174 (3)	−0.0009 (3)	0.0065 (2)	0.0002 (3)
C19	0.0170 (3)	0.0284 (3)	0.0159 (3)	−0.0006 (3)	0.0074 (2)	0.0004 (2)
C20	0.0160 (3)	0.0176 (3)	0.0159 (3)	0.0011 (2)	0.0048 (2)	−0.0010 (2)
C21	0.0181 (3)	0.0161 (3)	0.0193 (3)	−0.0016 (2)	0.0065 (2)	0.0001 (2)
C22	0.0188 (3)	0.0163 (3)	0.0176 (3)	0.0010 (2)	0.0071 (2)	0.0018 (2)
C23	0.0225 (4)	0.0423 (5)	0.0169 (3)	−0.0023 (3)	0.0040 (3)	0.0039 (3)

### Geometric parameters (Å, º)

**Table:**

O1—C1	1.3558 (9)	C10—H10A	0.9300
O1—C16	1.4613 (9)	C11—C12	1.3890 (11)
O2—C14	1.3543 (9)	C11—H11A	0.9300
O2—C15	1.4429 (9)	C12—C13	1.3971 (10)
O3—C1	1.2058 (9)	C12—H12A	0.9300
O4—C14	1.2073 (9)	C13—C14	1.4901 (10)
O5—C20	1.3633 (9)	C15—C16	1.5281 (11)
O5—C23	1.4303 (11)	C15—H15A	0.9700
C1—C2	1.4948 (9)	C15—H15B	0.9700
C2—C3	1.3955 (10)	C16—C17	1.5019 (10)
C2—C7	1.4080 (9)	C16—H16A	0.9800
C3—C4	1.3891 (10)	C17—C18	1.3946 (11)
C3—H3A	0.9300	C17—C22	1.3950 (10)
C4—C5	1.3864 (11)	C18—C19	1.3928 (11)
C4—H4A	0.9300	C18—H18A	0.9300
C5—C6	1.3913 (10)	C19—C20	1.3925 (11)
C5—H5A	0.9300	C19—H19A	0.9300
C6—C7	1.4016 (9)	C20—C21	1.3954 (11)
C6—H6A	0.9300	C21—C22	1.3846 (10)
C7—C8	1.4928 (10)	C21—H21A	0.9300
C8—C9	1.3995 (10)	C22—H22A	0.9300
C8—C13	1.4074 (10)	C23—H23A	0.9600
C9—C10	1.3929 (11)	C23—H23B	0.9600
C9—H9A	0.9300	C23—H23C	0.9600
C10—C11	1.3893 (12)		
			
C1—O1—C16	118.28 (6)	C8—C13—C14	120.98 (6)
C14—O2—C15	116.31 (6)	O4—C14—O2	125.25 (7)
C20—O5—C23	116.88 (6)	O4—C14—C13	124.78 (7)
O3—C1—O1	125.52 (6)	O2—C14—C13	109.97 (6)
O3—C1—C2	126.03 (6)	O2—C15—C16	108.98 (6)
O1—C1—C2	108.45 (6)	O2—C15—H15A	109.9
C3—C2—C7	120.66 (6)	C16—C15—H15A	109.9
C3—C2—C1	116.65 (6)	O2—C15—H15B	109.9
C7—C2—C1	122.68 (6)	C16—C15—H15B	109.9
C4—C3—C2	120.67 (7)	H15A—C15—H15B	108.3
C4—C3—H3A	119.7	O1—C16—C17	108.00 (6)
C2—C3—H3A	119.7	O1—C16—C15	107.09 (6)
C5—C4—C3	119.48 (7)	C17—C16—C15	111.98 (6)
C5—C4—H4A	120.3	O1—C16—H16A	109.9
C3—C4—H4A	120.3	C17—C16—H16A	109.9
C4—C5—C6	119.97 (7)	C15—C16—H16A	109.9
C4—C5—H5A	120.0	C18—C17—C22	118.59 (7)
C6—C5—H5A	120.0	C18—C17—C16	121.19 (7)
C5—C6—C7	121.80 (7)	C22—C17—C16	120.22 (7)
C5—C6—H6A	119.1	C19—C18—C17	121.36 (7)
C7—C6—H6A	119.1	C19—C18—H18A	119.3
C6—C7—C2	117.39 (6)	C17—C18—H18A	119.3
C6—C7—C8	118.10 (6)	C20—C19—C18	119.15 (7)
C2—C7—C8	124.49 (6)	C20—C19—H19A	120.4
C9—C8—C13	117.54 (6)	C18—C19—H19A	120.4
C9—C8—C7	118.09 (6)	O5—C20—C19	124.16 (7)
C13—C8—C7	124.37 (6)	O5—C20—C21	115.78 (7)
C10—C9—C8	121.61 (7)	C19—C20—C21	120.06 (7)
C10—C9—H9A	119.2	C22—C21—C20	120.11 (7)
C8—C9—H9A	119.2	C22—C21—H21A	119.9
C11—C10—C9	119.89 (7)	C20—C21—H21A	119.9
C11—C10—H10A	120.1	C21—C22—C17	120.71 (7)
C9—C10—H10A	120.1	C21—C22—H22A	119.6
C12—C11—C10	119.76 (7)	C17—C22—H22A	119.6
C12—C11—H11A	120.1	O5—C23—H23A	109.5
C10—C11—H11A	120.1	O5—C23—H23B	109.5
C11—C12—C13	120.18 (7)	H23A—C23—H23B	109.5
C11—C12—H12A	119.9	O5—C23—H23C	109.5
C13—C12—H12A	119.9	H23A—C23—H23C	109.5
C12—C13—C8	120.90 (6)	H23B—C23—H23C	109.5
C12—C13—C14	118.09 (6)		
			
C16—O1—C1—O3	−15.26 (10)	C9—C8—C13—C14	−174.01 (6)
C16—O1—C1—C2	165.24 (6)	C7—C8—C13—C14	6.70 (10)
O3—C1—C2—C3	−115.76 (8)	C15—O2—C14—O4	−15.77 (10)
O1—C1—C2—C3	63.74 (8)	C15—O2—C14—C13	163.90 (6)
O3—C1—C2—C7	64.78 (10)	C12—C13—C14—O4	−122.73 (8)
O1—C1—C2—C7	−115.72 (7)	C8—C13—C14—O4	55.03 (10)
C7—C2—C3—C4	−1.00 (10)	C12—C13—C14—O2	57.60 (8)
C1—C2—C3—C4	179.52 (6)	C8—C13—C14—O2	−124.64 (7)
C2—C3—C4—C5	1.90 (11)	C14—O2—C15—C16	−89.13 (8)
C3—C4—C5—C6	−1.03 (11)	C1—O1—C16—C17	149.64 (6)
C4—C5—C6—C7	−0.75 (11)	C1—O1—C16—C15	−89.59 (7)
C5—C6—C7—C2	1.62 (10)	O2—C15—C16—O1	52.88 (8)
C5—C6—C7—C8	−176.89 (6)	O2—C15—C16—C17	171.09 (6)
C3—C2—C7—C6	−0.74 (10)	O1—C16—C17—C18	66.97 (9)
C1—C2—C7—C6	178.70 (6)	C15—C16—C17—C18	−50.70 (9)
C3—C2—C7—C8	177.67 (6)	O1—C16—C17—C22	−112.89 (7)
C1—C2—C7—C8	−2.89 (10)	C15—C16—C17—C22	129.45 (7)
C6—C7—C8—C9	45.82 (9)	C22—C17—C18—C19	−1.10 (12)
C2—C7—C8—C9	−132.57 (7)	C16—C17—C18—C19	179.04 (7)
C6—C7—C8—C13	−134.90 (7)	C17—C18—C19—C20	0.54 (12)
C2—C7—C8—C13	46.71 (10)	C23—O5—C20—C19	8.11 (11)
C13—C8—C9—C10	−1.31 (10)	C23—O5—C20—C21	−171.16 (7)
C7—C8—C9—C10	178.02 (6)	C18—C19—C20—O5	−178.66 (7)
C8—C9—C10—C11	−1.62 (11)	C18—C19—C20—C21	0.58 (12)
C9—C10—C11—C12	2.22 (11)	O5—C20—C21—C22	178.19 (7)
C10—C11—C12—C13	0.14 (11)	C19—C20—C21—C22	−1.11 (11)
C11—C12—C13—C8	−3.16 (11)	C20—C21—C22—C17	0.54 (11)
C11—C12—C13—C14	174.61 (7)	C18—C17—C22—C21	0.55 (11)
C9—C8—C13—C12	3.69 (10)	C16—C17—C22—C21	−179.59 (6)
C7—C8—C13—C12	−175.60 (6)		

### Hydrogen-bond geometry (Å, º)

**Table:**

*D*—H···*A*	*D*—H	H···*A*	*D*···*A*	*D*—H···*A*
C16—H16*A*···O3^i^	0.98	2.60	3.2676 (10)	126

Symmetry code: (i) −*x*+1, −*y*+1, −*z*.
